# Concurrent Computed Tomography Angiography Spot Signs in Cerebral Contusion and Subdural Hematoma Following Severe Traumatic Brain Injury: A Case Report

**DOI:** 10.7759/cureus.96331

**Published:** 2025-11-07

**Authors:** Edgar G Ordóñez-Rubiano, Javier Romero, Isabella Lacouture

**Affiliations:** 1 Neurosurgery, Fundación Universitaria de Ciencias de la Salud, Bogota, COL; 2 Neurosurgery, Fundación Santa Fe de Bogotá, Bogota, COL; 3 Radiology/Neuroradiology, Massachusetts General Hospital, Boston, USA; 4 Neurological Surgery, Pontificia Universidad Javeriana, Bogota, COL

**Keywords:** advances in traumatic brain injury management, brain trauma injury, computed tomography angiography, spot sign, traumatic cerebral hematoma

## Abstract

The spot sign is a well-established radiologic marker associated with hematoma expansion and poor clinical outcomes in spontaneous intracerebral hemorrhage. However, its role in traumatic intracranial hemorrhages remains insufficiently characterized. We report the case of a 67-year-old man who sustained a severe traumatic brain injury following a collision with a bicycle-driven cart. On presentation, his Glasgow Coma Scale score was 3T, with anisocoria (right pupil 1.5 mm; left pupil 4 mm). Computed tomography angiography revealed concurrent spot signs in a left-sided acute subdural hematoma (SDH) and a right temporal contusion. Emergent left decompressive craniectomy was performed for the SDH. Due to progressive hematoma expansion, a contralateral craniectomy was subsequently required for the temporal contusion. This case highlights the rare concurrence of spot signs in two distinct traumatic intracranial hemorrhages. Recognition of this imaging feature may enhance prognostication and guide urgent neurosurgical interventions in traumatic brain injury with multiple traumatic intracranial hemorrhages.

## Introduction

Intracerebral hemorrhage (ICH) remains a leading cause of stroke-related morbidity and mortality. Although hematoma volume and location are critical predictors of clinical outcome, they are immutable at diagnosis. Since the 1980s, ICH-related hospitalization rates in the United States have remained steady at approximately 20 cases per 100,000 individuals annually [1]. However, the global incidence surged by 47% between 1990 and 2010, largely driven by rising rates in low-income countries. Despite therapeutic advances, hematoma expansion occurs in up to 40% of patients, significantly worsening prognosis, yet representing a potentially modifiable target for intervention [2].

The spot sign is a well-established radiologic marker that aids in predicting hematoma expansion and clinical outcomes in patients with ICH. Its clinical appeal lies in the fact that it can be readily identified through computed tomography angiography (CTA), a rapid, non-invasive imaging technique commonly used in acute stroke settings [3]. The presence of a spot sign correlates strongly with early neurological deterioration, typically defined as a ≥2-point drop in the Glasgow Coma Scale (GCS) or a ≥4-point rise in the National Institutes of Health Stroke Scale (NIHSS) in cases of secondary ICH [4]. Timely identification of this marker may play a pivotal role in guiding acute management and triaging patients to higher levels of care.

While the spot sign has been extensively characterized in the context of spontaneous ICH, its presence in traumatic brain injury (TBI) remains less well explored. Few existing literature studies discuss spot signs in TBI; few studies have suggested that the spot sign on computed tomographic angiography (CTA) is a sensitive radiological predictor of hematoma [5]. The presence of a spot and tail sign, assumed to indicate active bleeding from the striate artery, could be a more sensitive predictor of acute deterioration than the presence of a simple spot sign; considering this, computed tomography angiography (CTA) can be a standard imaging technique for severe TBI cases. A modified spot sign has been described recently, in which delayed-phase CT was performed 2 to 5 minutes after the first CT. The sensitivity and specificity were higher with the evaluation of the delayed-phase CT. However, these studies did not report the specific time of the second CT or the definition of a positive finding [6]. We present an illustrative case highlighting concurrent CTA spot signs; the uniqueness of this case is the presence of both a traumatic cerebral contusion and an acute subdural hematoma (SDH).

## Case presentation

Clinical findings

A 67-year-old man was admitted to the emergency department with a severe acute head trauma caused by an accident with a bicycle-driven cart. On examination, the following vital signs were recorded: blood pressure of 152/80 mm Hg, pulse of 79 beats per minute, respiratory rate of 20 breaths per minute, and oxygen saturation of 100% while intubated. The patient rated 3T on the Glasgow Coma Scale. He was not opening his eyes and had no verbal output but was moving all his extremities spontaneously. The right pupil was 1.5 mm in diameter (constricting to 1 mm to light), and the left pupil was 4 mm in diameter (fixed). He had clear stigmata of head trauma with epistaxis and a right proptotic eye.

Laboratory findings and outcomes in the ICU**

The prothrombin time (PT) was 12.0 seconds, which falls within the typical reference range of 11 to 14 seconds. INR was 1.0, consistent with the normal range of 0.8 to 1.2 for individuals not receiving anticoagulant therapy. The activated partial thromboplastin time (APTT) measured 30.7 seconds, which is within the expected range of 25 to 35 seconds. The platelet count was 316 × 10⁹/L (or 316,000/µL), also within the normal reference range of 150 to 450 × 10⁹/L. The patient required 18U STAT preoperative transfusion of platelets for aspirin use. Non-contrast computed tomography (NCCT) and CTA were performed at admission. A right SDH and an extensive subarachnoid hemorrhage (SAH) were noted, as well as multifocal cortical contusions involving the right parietal, bilateral frontal, and bilateral temporal lobes (see findings ahead). Emergent left decompressive craniectomy was performed for his acute SDH, and contralateral decompressive craniectomy was performed for his right temporal contusion as well. His OR course was remarkable for extremely labile blood pressure. The rest of his hospitalization was complicated by seizures, which were treated with propofol and subsequently by Keppra and valproic acid. The patient was initially managed in the Surgical Intensive Care Unit, as he required continuous critical care and monitoring; he was transferred to the Respiratory Acute Care Unit. Following discussions with the primary care and numerous consulting teams, comfort measures were provided. The patient deceased 22 days after admission.

Neuroimaging findings

**Figure 1:**
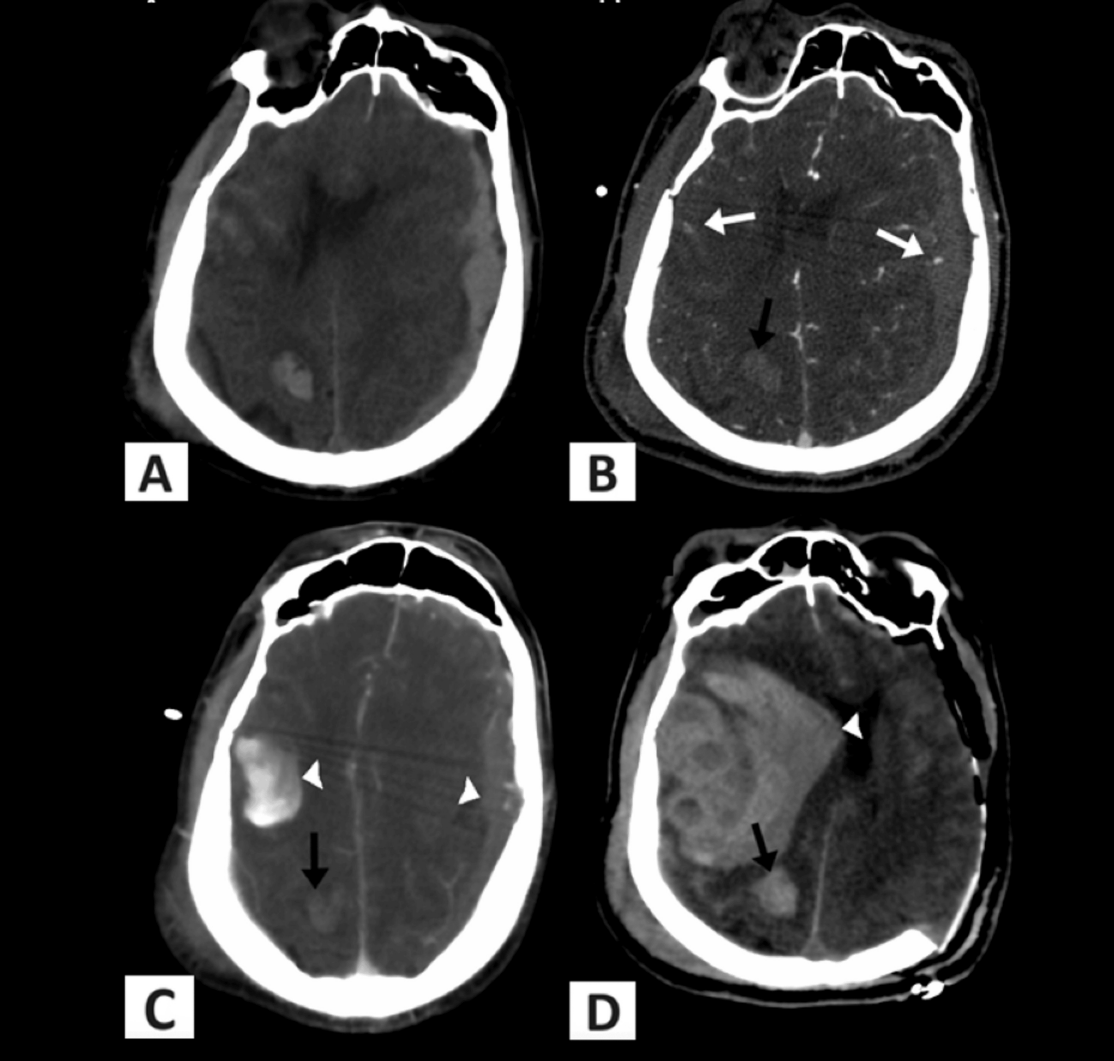
Pre- and postop neuroimaging (A) The initial NCCT of the head shows a large left convexity mixed density acute SDH, resulting in a mass effect on the underlying brain, with a rightward midline shift, and subfalcine herniation. Extensive SAH and multifocal cortical contusions involving the right parietal, bilateral frontal, and temporal lobes are noted. (B) The baseline CTA shows a left SDH and a right temporal contusion with two small foci of contrast extravasation consistent with spot signs (white arrows). It also demonstrates a right parietal contusion without a spot sign (black arrow). (C) The 90-second delay CTA shows marked expansion of both the right temporal contusion and of the left convexity SDH (arrowheads), but with no right parietal contusion expansion (black arrow). (D) Immediate postoperative NCCT demonstrates the increased size of the left frontal contusion (arrowhead) as predicted by the spot sign. There is no SDH in the left convexity due to the patient’s history of craniotomy. No parietal contusion expansion is noted (black arrow). NCCT: Non-contrast computed tomography; SDH: subdural hematoma; CTA: Computed tomography angiography.

The admission NCCT of the head showed a large left convexity mixed density acute SDH, resulting in mass effect on the underlying brain, with a 13 mm rightward midline shift, and subfalcine herniation. Extensive SAH was noted, as well as multifocal cortical contusions involving the right parietal, bilateral frontal and bilateral temporal lobes (Figure 1A). The first CTA showed two small foci of contrast pooling within a right temporal lobe contusion and a left SDH consistent with spot signs (Figure 1B). A subsequent 90-second delay CTA showed an increase in the size of the contusion, a SDH with substantial mass effect, and a rightward midline shift (Figure 1C). A follow-up CT confirmed further hematoma expansion with mass effect and a rightward midline shift (Figure 1D). Furthermore, the CT scans showed another contusion without a spot sign that did not have further hematoma expansion. 

Estimation of hematoma expansion was performed with the ABC/2 method. We found that the spot sign increased 30 times on the 90-second delay CTA, and the right frontal contusion expanded 737 times on the follow-up NCCT, compared with volume on the first CTA and first NCCT respectively (Table 1).

**Table 1:** ABC/2 method for estimation of hematoma expansion There was no SDH follow-up due to emergent left decompressive craniectomy. NCCT: Non-contrast computed tomography; CTA: Computed tomography angiography; ABC/2: performed method for estimating hematoma expansion; SDH: subdural hematoma; NA: unavailable.

	Admission Imaging ABC/2 (cm^3^)	Follow-Up Imaging ABC/2 (cm^3^) (Percent Increase)
NCCT		
Right Frontal Contusion	0.252	185.90 (73768%)
Right Parietal Contusion	2.97	5.38 (181%)
Left SDH	764.58	NA
CTA (Spot Sign Volume)		
Right Frontal Contusion	0.61	18.55 (3037%)
Right Parietal Contusion	0	0
Left SDH†	824.34	NA

Ethical approval and consent

The patient individually and voluntarily agreed to participate in the study after being fully informed about it. 

## Discussion

The spot sign is the presence of active contrast extravasation into primary non-traumatic ICH detected by computed tomography angiography [4]. It is an indicator of active hemorrhage and is associated with an increased risk of hematoma expansion, poor clinical outcome, and mortality in patients with primary ICH [7,8]. The spot sign, or contrast extravasation into the hematoma, is a reliable predictor of hematoma expansion, clinical deterioration, and increased mortality. Multiple studies have demonstrated a high negative predictive value for ICH expansion in patients without a spot sign [9]. However, while most literature focuses on primary ICH, this case supports its relevance in traumatic intracranial hemorrhage, where hematoma expansion is also a critical determinant of prognosis. 

Various radiological risk factors have been identified for hematoma expansion, including a larger initial hematoma size, the concurrent presence of SAH, and/or SDH. Clinical factors that predict hematoma expansion are advanced age, hypertension, and the presence of coagulopathy [10]. Some studies have shown that effects of cerebral injury attributed to trauma and ICH are similar [11]. However, the mechanisms of hemorrhage progression and the association of the spot sign with TBI have not been entirely elucidated [12]^_._^

The spot sign has a moderate sensitivity (62%) and a high specificity (88%) for predicting ICH. Interestingly, a delayed CTA spot sign showed a much higher sensitivity (79%) and a slightly lower specificity (84%) for predicting ICH. It is important to note that the presence of the dynamic spot sign within the hematoma may reflect ongoing bleeding, and most of the spot signs occurred in the arterial phase, suggesting small artery damage and bleeding [13]. The presence of a spot sign is a common finding and is associated with longer hospital stay and possibly worse functional outcomes in TBI survivors [14]. The limitations of the spot sign include the need for an early CTA examination, the growth in hematoma volume occurs in approximately one-third of patients with acute ICH within six hours and is associated with early neurologic deterioration and functional outcome, which is not readily available in many institutions and its prevalence decreases with delayed imaging [15]. 

Despite emergent bilateral decompressive craniectomies, the patient experienced ongoing seizures and a poor outcome, consistent with the high mortality observed in patients with spot sign-positive hemorrhage. The extravasation of contrast in the setting of traumatic brain contusion is a useful imaging sign to predict hematoma expansion, worse neurological outcomes, and higher mortality. Considering the low number of studies and limited sample sizes, additional multi-center, adequately powered studies with more standardized methods are needed to the validation of this hypothesis and wider use, if appropriate [16]. 

The study shows limitations such as the lack of follow-up data on functional outcomes. By being a single case study, there is an inability to generalize clinical findings. Despite not being unprecedented, this case acknowledges concurrent spot signs being meaningful in trauma, which is under reported in the literature. This case demonstrates the potential value of the spot sign in traumatic brain injury as an imaging biomarker of hematoma expansion and the importance of doing more research to understand the pathophysiologic mechanisms and their determining factors. 

## Conclusions

The spot sign is a well-established radiologic marker that aids in predicting hematoma expansion and clinical outcomes in patients with ICH. The results of this case are significant in that they shed light on the importance of the spot sign in the probable prediction of hematoma expansion in patients with TBI. This phenomenon has not been previously described in patients with TBI in published literature to our knowledge. Furthermore, it is necessary to systematically characterize the CTA spot sign in a large TBI patient population, as well as evaluate if it is associated with an increased risk of hematoma expansion, poor clinical outcome, or mortality in patients with TBI. This radiological marker may be useful in the evaluation of patients with TBI who are experiencing neurologic decline and may need surgical treatment.
